# Supplementary material for: Incidence and factors associated with reoperation after rotator cuff repair in Korea: A nationwide cohort study
Source: PLoS One. 2026 May 26;21(5):e0350201. doi: 10.1371/journal.pone.0350201 (PMC13210397; doi:10.1371/journal.pone.0350201)
Supplement: Supplementary Table 1 — (DOCX) [file pone.0350201.s001.docx]

**Supplementary Table 1. International Classification of Diseases, 10th revision of shoulder-related disease**

| **ICD-10** | **Description (Detailed ICD-codes)** |
| --- | --- |
| M13 | Other arthritis (M130, M1311, M1312, M1319, M1380, M1381, M1382, M1389, M1390, M1391, M1392, M1399) |
| M19 | Other arthrosis (M1900, M1901, M1902, M1909, M1910, M1911, M1912, M1919, M1920, M1921, M1922, M1929, M1980, M1981, M1982, M1989, M1990, M1991, M1992, M1999) |
| M24 | Other specific joint derangements (M2400, M2401, M2402, M2409, M2410, M2411, M2412, M2419, M2430, M2431, M2432, M2439, M2440, M2441, M2442, M2449, M2450, M2451, M2452, M2459, M2460, M2461, M2462, M2469, M2470, M2479, M2480, M2481, M2482, M2489, M2490, M2491, M2492, M2499) |
| M25 | Other joint disorders, NEC (M2500, M2501, M2502, M2509, M2510, M2511, M2512, M2519, M2520, M2521, M2522, M2529, M2530, M2531, M2532, M2539, M2540, M2541, M2542, M2549, M2550, M2551, M2552, M2559, M2560, M2561, M2562, M2569, M2570, M2571, M2572, M2579, M2580, M2581, M2582, M2589, M2590, M2591, M2592, M2599) |
| M60 | Myositis (M6000, M6001, M6002, M6009, M6010, M6011, M6012, M6019, M6020, M6021, M6022, M6029, M6080, M6081, M6082, M6089, M6090, M6091, M6092, M6099) |
| M62 | Other disorders of muscle (M6200, M6201, M6202, M6209, M6210, M6211, M6212, M6219, M6220, M6221, M6222, M6229, M6230, M6231, M6232, M6239, M6240, M6241, M6242, M6249, M6250, M6251, M6252, M6259, M6260, M6261, M6262, M6269, M6280, M6281, M6282, M6289, M6290, M6291, M6292, M6299) |
| M65 | Synovitis and tenosynovitis (M6500, M6501, M6502, M6509, M6510, M6511, M6512, M6519, M6520, M6521, M6522, M6529, M6530, M6531, M6532, M6539, M6580, M6581, M6582, M6589, M6590, M6591, M6592, M6599) |
| M67 | Other disorders of synovium and tendon (M6710, M6711, M6712, M6719, M6720, M6721, M6722, M6729, M6730, M6731, M6732, M6739, M6740, M6741, M6742, M6749, M6780, M6781, M6782, M6789, M6790, M6791, M6792, M6799) |
| M70 | Soft tissue disorders related to use, overuse and pressure (M702) |
| M72 | Fibroblastic disorders (M7240, M7241, M7242, M7249, M7260, M7261, M7262, M7269, M7280, M7281, M7282, M7289, M7290, M7291, M7292, M7299) |
| M75 | Shoulder lesions (M750, M751, M752, M753, M754, M755, M756, M758, M759) |
| M79 | Other soft tissue disorders, NEC (M7900, M7901, M7902, M7909, M7910, M7911, M7912, M7919, M7920, M7921, M7922, M7929, M7930, M7931, M7932, M7939, M7950, M7951, M7952, M7959, M7960, M7962, M7969, M7970, M7971, M7972, M7979, M7980, M7981, M7982, M7989, M7990, M7991, M7992, M7999) |
| S43 | Dislocation, sprain and strain of joints and ligaments of shoulder girdle (S4300, S4301, S4302, S4308, S4309, S431, S432, S433, S434, S435, S436, S437) |
| S44 | Injury of nerves at shoulder and upper arm level (S440, S441, S442, S443, S444, S445, S447, S448, S449) |
| S46 | Injury of muscle and tendon at shoulder and upper arm level (S4600, S4608, S4610, S4618, S4620, S4628, S4630, S4638, S4670, S4678, S4680, S4688, S4690, S4698) |
| S49 | Other and unspecified injuries of shoulder and upper arm (S497, S498, S499) |
