# Supplementary material for: Incidence and factors associated with reoperation after rotator cuff repair in Korea: A nationwide cohort study
Source: PLoS One. 2026 May 26;21(5):e0350201. doi: 10.1371/journal.pone.0350201 (PMC13210397; doi:10.1371/journal.pone.0350201)
Supplement: Attachment — Submitted filename: Response to Reviewer_final.docx [file pone.0350201.s004.docx]

Response to Reviewer’s comments

**Reviewer #1:**

**1. The author uses surgical codes N0935–N0938 to define whether revision surgery was performed. Among them, N0935 represents simple acromioplasty, which can be done independently without tendon re-tear. Whether it can be equated with repair surgery remains to be verified. This may introduce many cases that do not fall under "repair failure", such as when the tendon heals well after surgery but there is still subacromial impingement pain, and simple acromioplasty is chosen to relieve pressure and pain. This may lead to an overestimation of the "reoperation rate" and increase confounding factors. It is suggested that in addition to the current analysis, a sensitivity analysis using only patients with codes N0936–N0938 be added as supplementary material.**

Response:

Thank you for this valuable and thoughtful comment. We fully agree with the reviewer that simple acromioplasty (N0935) does not necessarily reflect structural failure of the repaired tendon and may therefore contribute to a modest overestimation of the reoperation rate. We also agree that a sensitivity analysis restricted to N0936–N0938 would further clarify the robustness of our findings.

In our study, however, the outcome was intentionally defined as reoperation, rather than structural re-tear, because claims data do not allow differentiation between tendon failure and other postoperative indications. Procedural codes do not capture imaging findings, intraoperative assessments, or surgical intent; thus, reoperations performed for persistent pain, impingement, or other clinical causes cannot be distinguished from those performed for true re-tear. For this reason, we used N0935–N0938 to represent the full spectrum of repeat operative management after the index RCR. To address this concern clearly, we have strengthened the operational definition of reoperation in the Methods section by explicitly describing reoperation as any subsequent surgical intervention following the index repair and by clarifying the use and validation of these codes. To address this issue more explicitly in the manuscript, we added the following clarifying sentence to the Discussion: “Because procedural codes cannot distinguish true re-tears from other postoperative indications, the outcome was intentionally defined as reoperation to capture all clinically relevant repeat surgeries after the index repair.” (Line 404-406) We have also noted the possibility that the inclusion of N0935 may slightly overestimate the absolute reoperation rate.

In addition, because the reviewer’s observation suggested that some expressions in the original manuscript could be interpreted as referring to structural re-tear, we carefully reviewed and revised the wording throughout the manuscript. All outcome-related terminology has now been standardized to explicitly state “reoperation”, ensuring that the intended scope of the study is consistently communicated and that any potential confusion between reoperation and re-tear is avoided.

Although we agree with the reviewer’s suggestion, we are unable to perform additional analyses at this stage. The HIRA research data used in this study were accessible only through a restricted remote-analysis environment during a predefined analysis window. After the approved period ended, the analysis environment was closed under HIRA policy, and no further access or reanalysis is permitted.

To address the reviewer’s concern to the extent possible within these constraints, we have added an alternative supplementary sensitivity analysis that was conducted during the original analysis period. In this analysis, the Charlson Comorbidity Index categories used in the main model (0, 1, 2, and ≥3) were reclassified into a dichotomous form (0 vs. ≥1) to examine the stability of the estimated associations. The resulting hazard ratio estimates remained consistent with those of the primary model, supporting the robustness of our findings. This analysis has been added as Supplementary Table 1, and the corresponding descriptions have been incorporated into the Methods and Results sections as follows:

“In addition to the main multivariate Cox proportional hazards model, we conducted a supplementary sensitivity analysis in which the original four CCI categories (0, 1, 2, and ≥3) were reclassified into a dichotomous variable (0 vs. ≥1) to assess the stability of the estimated associations.” (Lines 211–214)

“The supplementary sensitivity analysis using a dichotomized CCI (0 vs. ≥1) yielded hazard ratio estimates that were consistent with those of the main model, indicating that the overall findings were robust (Supplementary Table 1).” (Lines 315–317)

We sincerely appreciate the reviewer’s insightful suggestion, which has helped us strengthen the clarity of our outcome definition and improve the overall transparency of the manuscript.

**2. Does the HIRA data include information on the affected side? Is it possible that surgeries on the contralateral shoulder of the same patient during the follow-up period were counted as revisions? This should be explained in the limitations section.**

Response:

Thank you for this important comment. We fully agree that laterality (left/right shoulder information) is an essential factor when interpreting reoperation rates. The HIRA claims database does not provide laterality details for surgical procedures; therefore, we could not determine whether subsequent RCR procedures were performed on the ipsilateral or contralateral shoulder. As the reviewer pointed out, this raises the possibility that contralateral shoulder surgeries may have been counted as revision surgeries.

To address this issue, we have added a clear explanation of this limitation in the revised manuscript. Specifically, we have incorporated the following sentence into the Limitations section:

“Second, the HIRA database does not provide laterality information, so we could not fully determine whether follow-up procedures occurred on the same shoulder; therefore, some contralateral surgeries may have been counted as reoperations and should be taken into consideration when interpreting the results.” (Line 398-401)

We appreciate the reviewer’s insightful suggestion, which has helped improve the clarity and interpretability of our study.

**3. The study limited the observation period to 6 months to 3.5 years after surgery, excluding reoperations within 6 months. The author acknowledges in the discussion that this may underestimate the incidence rate. However, the fundamental problem with this approach is that early failures are systematically excluded, making the sample selection more "healthy" and potentially leading to selection bias. It is suggested to include a sensitivity analysis covering the 0–3.5-year follow-up period, or at least provide the event rate for 0–6 months to allow readers to judge the extent of underestimation.**

Response:

Thank you for this insightful comment. We agree that excluding reoperations within the first 6 months may lead to an underestimation of the overall reoperation incidence and could introduce a degree of selection bias, as early failures are not captured in our analysis.

However, early reoperations during this period are generally attributable to technical issues or acute surgical complications rather than the patient-, procedure-, or postoperative management factors that our study aimed to evaluate. In addition, reoperations occurring within the first 6 months overlap with the period in which key postoperative management variables are measured. Because postoperative factors are assessed during this same window, including early reoperations as outcomes would create temporal ambiguity and a risk of reverse causation, making it analytically inappropriate to evaluate their association with postoperative factors. Our primary analytic objective was to examine pre-, intra-, and postoperative factors—including postoperative management provided during the initial 6 months—in relation to mid- to long-term outcomes. For this reason, we defined the reoperation outcome from 6 months onward to ensure that early technical failures were not conflated with factors of interest. Therefore, restricting the outcome window to 6–3.5 years was necessary to align the outcome definition with our study purpose.

We fully acknowledge the reviewer’s concern and have clarified this in the revised manuscript. Specifically, we added the following explanation to the Limitations section:

“Fourth, because the follow-up period was defined from 6 months to 3.5 years after surgery, early reoperations within the initial 6 months were not captured. We acknowledge that this may lead to an underestimation of the overall reoperation incidence and may introduce a degree of selection bias. However, early reoperations are generally related to technical issues or acute surgical complications, while our study aimed to evaluate pre-, intra-, and postoperative factors—including postoperative management provided during the initial 6 months—in relation to mid- to long-term outcomes. Therefore, excluding early events was necessary to align the outcome definition with the objective of our analysis.” (Line 406-413)

We appreciate the reviewer’s helpful suggestion, which has allowed us to improve the clarity of our study design and limitations.

Ref) 1. Mandaleson, Avanthi. "Re-tears after rotator cuff repair: current concepts review." *Journal of clinical orthopaedics and trauma* 19 (2021): 168-174.

2. Felsch, Quinten, et al. "Complications within 6 months after arthroscopic rotator cuff repair: registry-based evaluation according to a core event set and severity grading." *Arthroscopy: The Journal of Arthroscopic & Related Surgery* 37.1 (2021): 50-58.

**4. The information page and the main text have inconsistent statements regarding "Funding". The information page states "No specific funding", while the main text lists KSN2121211. Please unify them.**

Response:

Thank you for pointing this out. We apologize for the inconsistency between the information page and the main text regarding the funding source. This study was supported by the Korea Institute of Oriental Medicine (project number KSN2121211), and the correct funding information should appear consistently throughout the manuscript.

We have revised the information page to match the main text and now clearly state the appropriate funding source (KSN2121211). The corrected information has been updated in the revised submission.

We appreciate the reviewer’s careful attention to detail.

**Reviewer #2: 

1. Clarify the definition and validation of “reoperation” and discuss possible underestimation from excluding early failures (<6 months).**

Response:

Thank you for this constructive comment. In our study, “reoperation” was defined as any subsequent surgical intervention performed to manage recurrent or persistent rotator cuff pathology after the index repair. Patients who underwent reoperation were identified as those who received subsequent rotator cuff procedures coded as N0935, N0936, N0937, or N0938 in the HIRA database. These procedural codes are standardized within the Korean National Health Insurance system and have been widely used in nationwide claims-based studies to identify rotator cuff surgeries, supporting their validity for use in administrative data research (Jo et al., 2017). To improve clarity, we have added this operational definition and the supporting reference to the Methods section of the revised manuscript.

“Reoperation was defined as any subsequent surgical intervention to manage recurrent or persistent rotator cuff pathology after the index repair. Patients who underwent reoperation were identified as those who received subsequent rotator cuff procedures coded as N0935, N0936, N0937, or N0938 after the primary RCR. These procedural codes are standardized within the Korean National Health Insurance system and have been used in nationwide claims-based studies to identify rotator cuff surgeries, supporting their validity for use in administrative data research” (Line 148-154)

Regarding the exclusion of early failures, reoperations occurring within the first 6 months were not captured because our follow-up window was defined from 6 months to 3.5 years after surgery. We acknowledge that this may lead to an underestimation of the overall reoperation incidence. Early reoperations are often attributable to technical issues or acute surgical complications, whereas our study aimed to evaluate pre-, intra-, and postoperative factors—including postoperative management during the initial 6 months— in relation to mid- to long-term outcomes. Therefore, excluding early events was necessary to align the outcome definition with the analytic objective of our study.

To clarify this point, we have revised the Limitations section in the manuscript and added the following sentence:

“Fourth, because the follow-up period was defined from 6 months to 3.5 years after surgery, early reoperations within the initial 6 months were not captured. We acknowledge that this may lead to an underestimation of the overall reoperation incidence and may introduce a degree of selection bias. However, early reoperations are generally related to technical issues or acute surgical complications, while our study aimed to evaluate pre-, intra-, and postoperative factors—including postoperative management provided during the initial 6 months—in relation to mid- to long-term outcomes. Therefore, excluding early events was necessary to align the outcome definition with the objective of our analysis.” (Line 406-413)

We appreciate the reviewer’s helpful suggestion, which improved the clarity and transparency of our methodology.

Ref)

1. Mandaleson, Avanthi. "Re-tears after rotator cuff repair: current concepts review." Journal of clinical orthopaedics and trauma 19 (2021): 168-174.

2. Felsch, Quinten, et al. "Complications within 6 months after arthroscopic rotator cuff repair: registry-based evaluation according to a core event set and severity grading." Arthroscopy: The Journal of Arthroscopic & Related Surgery 37.1 (2021): 50-58.

**2. Expand discussion on unmeasured confounders (tear morphology, surgical technique, surgeon factors).**

Response:

Thank you for this helpful comment. We agree that important unmeasured confounders, such as tear morphology, surgical technique, and surgeon-related factors, may influence retear and reoperation risk after rotator cuff repair. Because our study was based on administrative claims data, we were unable to obtain detailed information on tear size and pattern, tendon quality or fatty degeneration, specific repair configurations, augmentation procedures, surgeon experience, or operative time. Previous clinical and imaging studies have reported that tear morphology, tendon quality, surgical techniques, and surgeon factors are associated with healing, retear, and subsequent surgical outcomes after rotator cuff repair.

To address the reviewer’s suggestion, we have expanded the description of unmeasured confounders in the Limitations section as follows:

“Seventh, although we adjusted for covariates such as age, comorbidities, insurance type, and hospital type, we could not account for several important clinical and surgical factors that are not available in claims data, including tear size and pattern, tendon quality and fatty degeneration, detailed surgical techniques, surgeon experience, and operative time. Previous clinical and imaging studies have shown that tear morphology, tendon quality, surgical technique, and surgeon-related factors are associated with healing, retear, and reoperation risk after rotator cuff repair (11,16). Therefore, residual confounding by these unmeasured factors may remain.” (Line 418-425)

We appreciate the reviewer’s suggestion, which helped us clarify this important limitation more explicitly.

**3. Specify variables included in the multivariate model and confirm assumption testing.**

Response:

Thank you for this constructive suggestion. To improve clarity, we revised the Methods section to explicitly list the variables included as candidate covariates in the multivariate model.

We also refined our description of the proportional hazards assumption test. In the revised Methods section, we clarified that the proportional hazards assumption was assessed using Schoenfeld residuals and supplemented by visual inspection of log–log plots. The Results section has also been updated to include the findings from these assumption tests. Although a small number of variables showed borderline deviations in individual tests, the global test indicated no violation of the proportional hazards assumption, and the log–log plots suggested that the model met this assumption. A brief note was also added to the Discussion to indicate that these minor deviations are unlikely to influence the interpretation of our main findings.

The corresponding revisions to the manuscript are summarized below:

“Candidate risk factors for the multivariate model included most of preoperative, intraoperative, and postoperative factors described above, namely sex, age group, health insurance type, healthcare institution type, CCI score, type of surgery (N0935, N0936, N0937, or N0938), arthroscopy use, primary diagnosis, use of KMHC (including acupuncture, electroacupuncture, herbal medicine, rehabilitation therapy, and Korean medicinal physical therapy), timing to the first outpatient visit for KMHC, and timing to the first outpatient visit for CMHC.” (Line 204-210)

“The proportional hazards assumption was evaluated using Schoenfeld residuals and confirmed by visual inspection of log–log plots.” (Line 214-215)

“Although a few variables showed borderline deviations in the Schoenfeld residual tests, the global test indicated no violation of the proportional hazards assumption. The log–log plots also demonstrated approximately parallel curves across groups, supporting the adequacy of the proportional hazard assumption.” (Line 310-313)

“Sixth, although a few variables showed borderline deviations in the proportional hazards assumption test, these deviations were minor, and the global test and log–log plots indicated that the overall proportional hazards assumption was reasonably met.” (Line 415-418)

**4. Enhance figures/tables (confidence bands on survival curves, consistent CI formatting).**

Response:

Thank you for this thoughtful comment. To address the reviewer’s suggestion to enhance the figures and tables, we made two specific revisions. First, confidence intervals were added to Table 2 to incorporate confidence bands corresponding to the survival curve–based cumulative incidence estimates. The confidence intervals were formatted consistently with those presented in Table 3 (e.g., 1.31 (1.24-1.39)) to improve uniformity across the manuscript. Second, Figure 2 was regenerated as a high-resolution TIFF file (600 dpi) to enhance its visual clarity and ensure high-quality presentation of the hazard ratios and their confidence intervals. We believe these improvements strengthen both the readability and consistency of the figures and tables as requested.

**5. Streamline the Discussion to focus on the main findings and reduce redundancy.**

Response:

Thank you for this valuable suggestion. We carefully reviewed the Discussion section and revised it to improve clarity and focus. Redundant explanations were removed, overlapping content was condensed, and the narrative was reorganized to emphasize the study’s main findings, the implications of key results, and essential contextual interpretation. These revisions have resulted in a more concise and coherent Discussion section. We appreciate the reviewer’s guidance, which helped strengthen the manuscript.
