# Supplementary material for: Incidence and factors associated with reoperation after rotator cuff repair in Korea: A nationwide cohort study
Source: PLoS One. 2026 May 26;21(5):e0350201. doi: 10.1371/journal.pone.0350201 (PMC13210397; doi:10.1371/journal.pone.0350201)
Supplement: Attachment — Submitted filename: Response to Reviewers.docx [file pone.0350201.s005.docx]

**Response to Reviewers**

**Reviewer Comment 1**

**Comment:**

Definition of “Reoperation” vs. Structural Failure

The authors appropriately clarify that the outcome is reoperation, not structural re-tear, and acknowledge the limitations of claims data. However, despite these clarifications, parts of the Introduction and Discussion still implicitly frame the findings in the context of tendon failure and tear characteristics (e.g., references to tear size and structural complexity). Further tighten the language throughout the manuscript to consistently distinguish clinical reoperation from biological or structural failure. Statements implying causality between postoperative care and tendon integrity should be softened to reflect association rather than mechanism.

**Response:**

We thank the reviewer for this important and constructive comment. In response, we have carefully revised the manuscript to ensure a consistent distinction between clinical reoperation and structural or biological failure throughout the Introduction and Discussion sections.

**1) Clarification of outcome definition (Methods section)**

We clarified the definition of reoperation in the Methods section (Lines 152-153) as follows:

*Line 152-153: “Reoperation was defined as any subsequent surgical intervention performed after the index RCR, regardless of the underlying clinical indication.”*

This revision ensures that the outcome is explicitly defined as a clinical event and not a surrogate for structural failure.

**2) Removal of structural failure–oriented framing (Introduction and Discussion)**

We revised the Introduction and Discussion sections (Introduction: Lines 58-81; Discussion: Lines 327, 347, 389) to remove or rephrase statements that could implicitly link reoperation to structural failure or tendon re-tear.

*Line 58-67: “Despite the outstanding clinical outcomes of rotator cuff repair (RCR) and the evolving surgical techniques, postoperative adverse outcomes remain a concern. In particular, reoperation following RCR has been reported in a wide range of cases (11% to 94%), representing an important clinical event after surgery(8, 9). It should be noted that reoperation does not necessarily indicate structural failure or tendon re-tear, as not all structural failures lead to surgical intervention, and reoperations may occur for various clinical reasons. To date, factors associated with reoperation after RCR remain unclear. Preoperative characteristics such as patient factors, tear and shoulder morphology, intraoperative repair management, and postoperative rehabilitation strategies may be associated with the likelihood of reoperation(10, 11).”*

**3) Removal of causal language across the manuscript**

Throughout the manuscript, expressions implying causality (e.g., “influence,” “lead to,” “reduce”) were systematically replaced with neutral, association-based terms such as “associated with” or “may reflect.”

To further ensure consistency in the interpretation of our findings, we revised the manuscript title from:

*“Incidence and prognostic factors of reoperation after rotator cuff repair in Korea: A nationwide cohort study”*

to:

*“Incidence and factors associated with reoperation after rotator cuff repair in Korea: A nationwide cohort study.”*

This change reflects the observational nature of the study and avoids potential causal or predictive implications.

**Reviewer Comment 2**

**Comment:**

Inclusion of Acromioplasty (N0935) as Both Index and Outcome Procedure

Although the rationale for including N0935 is explained, this code represents heterogeneous clinical scenarios and may bias both incidence estimates and hazard ratios—particularly given that N0935 appears protective in multivariable analysis. Explicitly caution readers in the Discussion that hazard ratios associated with procedure codes should not be interpreted as causal effects. A short paragraph explaining potential confounding by indication would strengthen interpretability.

**Response:**

We thank the reviewer for this insightful comment. We agree that the inclusion of N0935 (acromioplasty) may reflect heterogeneous clinical scenarios and that the observed association should be interpreted with caution. In response, we have revised the Discussion section (Lines 361-367) to explicitly address potential confounding by indication. Specifically, we clarified that N0935 may be preferentially performed in patients with less severe disease or smaller tears, which may lead to an apparent protective association with reoperation. We further emphasized that hazard ratios associated with procedural codes should not be interpreted as causal effects, but rather as reflecting underlying differences in patient characteristics or disease severity. In addition, we noted that important clinical variables such as tear size, tendon quality, and surgical complexity are not available in the claims data, and therefore procedural codes may act as proxies for unmeasured severity, contributing to residual confounding.

*Line 361-367: However, this finding should be interpreted with caution, as N0935 may be preferentially performed in patients with less severe disease, leading to potential confounding by indication. Therefore, hazard ratios associated with procedural codes should not be interpreted as causal effects. These findings are consistent with prior evidence that initial tear size and structural complexity are major determinants of adverse structural outcomes reported in clinical studies, although such factors were not directly measured in the present claims-based analysis.*

**Reviewer Comment 3**

**Comment:**

Exclusion of Early Reoperations (<6 months)

The justification for excluding early reoperations is reasonable from an analytic standpoint. However, this design choice fundamentally alters the interpretation of the reported incidence. Emphasize more clearly (including in the Abstract or Discussion opening) that the reported 6.82% incidence reflects mid- to long-term reoperation, not overall surgical failure. This distinction is crucial for clinical readers.

**Response:**

We thank the reviewer for this important comment. We agree that excluding early reoperations affects the interpretation of the reported incidence. In response, we have revised both the Abstract (Lines 30-31) and the opening of the Discussion (Lines 337-338) to clearly state that the reported 6.82% incidence reflects mid- to long-term reoperation (from 6 months to 3.5 years after surgery), rather than overall surgical failure. We believe this clarification improves the clinical interpretability of our findings and aligns with the reviewer’s recommendation.

*Line 30-31, Line 337-338: Using the Korean National Health Insurance Database from 2011 to 2021, we identified a cumulative reoperation incidence of 6.82% during the follow-up period from 6 months to 3.5 years after primary repair, reflecting mid- to long-term reoperation rather than overall surgical failure.*
